# PReS-FINAL-2120: Juvenile scleroderma international network (JUSINET) database: a reliable instrument for clinical research in juvenile scleroderma syndromes

**DOI:** 10.1186/1546-0096-11-S2-P132

**Published:** 2013-12-05

**Authors:** G Cuffaro, G Martini, F Sperotto, T Avcin, R Russo, O Kasapcopur, F Vittadello, F Zulian

**Affiliations:** 1 Department of Pediatrics, University of Padua, Padua, Italy; 2 Department of Allergy, Rheumatology and Clinical Immunology, University Children's Hospital, University Medical Center Ljubljana, Ljubljana, Slovenia; 3 Hospital de Pediatria Juan P. Garrahan of Buenos Aires, Buenos Aires, Argentina; 4 Department of Pediatrics, Cerrahpasa Tip Fakultesi, Instanbul, Turkey

## Introduction

The conduct of Clinical Research in rare diseases, such as Juvenile Systemic Sclerosis (jssc) and Juvenile Localized Scleroderma (JLS), requires an adequate number of patients and a fruitful collaboration between international centers. The clinical management of young patients suffering from these diseases is also often difficult to achieve in an effective and shared matter.

## Objectives

We propose a web-based registry (http://www.jusinet.org) to prospectively collect data on demographic, epidemiological, clinical, and laboratory features of patients with jssc and JLS from adult and paediatric rheumatology centres and to educate physicians to a more standardized approach to these conditions.

The purpose is to provide a well-characterized cohort of scleroderma patients according to the current classification criteria and collect adequate information enabling to uniform clinical assessment and diagnostic tests, to stimulate clinical and basic research projects.

## Methods

The Database was evaluated by some international experts who provided us with valuable advice for improvement.

JUSINET has an administrative structure including a Database Executive Committee (DEC), who evaluates progress of the project and discuss management issues.

The Database Coordinator (DC) assisted by a Research Assistant (RA), and a Database Manager (DM, statistician) form the Local Administrative Structure (LAS).

In order to verify the performance of JUSINET at national and international level, four centers in Italy, one in Slovenia, Argentina and Turkey, have tested and validated the system including real patients cases. Compilers were required to express their opinion on 3 variables, clarity of information, ease of data entry and completeness of information, for each section of the database.

## Results

The 324 opinions expressed for the 22 sections of JUSINET, in a range between 1-5, reached a mean value of 4.62. The mean time to enter a new patient data was 14 minutes for jssc, and 8 minutes for JLS; to update data was 8 minutes for jssc and 5 minutes for JLS.

## Conclusion

The JUSINET Database represents a valuable instrument to better characterize patients childhood onset scleroderma and facilitate research on pathogenesis and treatment of this relatively rare condition. It also provides a simple and reliable tool for the daily clinical management of these patients.

## Disclosure of interest

None declared.

